# Response to: Comment on “Differences in Ventilatory Threshold for Exercise Prescription in Outpatient Diabetic and Sarcopenic Obese Subjects”

**DOI:** 10.1155/2017/7026597

**Published:** 2017-11-19

**Authors:** Gian Pietro Emerenziani, Maria Chiara Gallotta, Silvia Migliaccio, Emanuela A. Greco, Chiara Marocco, Luca di Lazzaro, Rachele Fornari, Andrea Lenzi, Carlo Baldari, Laura Guidetti

**Affiliations:** ^1^Department of Experimental and Clinical Medicine, University of Magna Graecia of Catanzaro, Catanzaro, Italy; ^2^Department of Movement, Human and Health Sciences, University of Rome “Foro Italico”, Rome, Italy; ^3^Department of Experimental Medicine, Sapienza University of Rome, Rome, Italy; ^4^LiSa Laboratory, Policlinico of Catania, University of Catania, Catania, Italy

We would like to thank Goran Kuvačić [1] for his interest to our study [2]. We included our answers below.

- In 2.2. Clinical Evaluation. Shimamoto et al. [3] studied seasonal variations in body composition and anthropometric characteristics during a weight loss program. The aim of our study was to examine cardiorespiratory parameters at IVT and peak exercise capacity, and it was not to evaluate the effects of a weight loss program on body composition in obese subjects. However, the season (January 2013 to June 2013) and time of the day (morning) are stated in the text, and the precise day and hour of the morning were randomly distributed between the three studied groups.
- In 2.2. Clinical Evaluation. Subjects were evaluated both by DEXA and by BIA and were included in the study when both %FFM values were above the declared limit. In addition, subjects were evaluated both by DEXA and BIA for other specific issues, such as bone mineral density and trabecular bone score, after 12 months of protocol which, however, were not part of the results included in the manuscript. These data can be obtained only by DEXA, and not by BIA, and were not included since they were not part of the specific aims of the study described herein.
- In 2.2. Clinical Evaluation. The aim of our study was not to evaluate the effects of diet on body composition on obese subjects. Subjects had received a diet program on the same day of the clinical evaluation, and they had to be on diet after the physical exercise test. They were enrolled to participate in a 4-month intervention protocol, which will be part of a further study.
- In 2.2. Clinical Evaluation. The methodology was chosen to specifically address whether differences could exist on physiological parameters at IVT and at maximal effort in the obese with sarcopenia or diabetes mellitus subjects. The “clinical evaluations” were not the outcomes of the study but were included to describe the subjects participating into the study.
- In 2.3. Maximal Effort and Individual Ventilatory Threshold. The citation regards the fact that maximal effort and IVT were assessed by maximal-graded exercise test on a treadmill according to individual abilities as previously done in another author's study [4]. The test protocol as described in the article is written in a concise manner that makes it possible to replay it: “Treadmill protocol started at 3 km/hand then speed increased by 1 km/h every two minutes until 5 km/h was reached. Then, slope was increased by 3% every two minutes until subjects reached a value of 10 on RPE scale.”
- In 2.3. Maximal Effort and Individual Ventilatory Threshold. It is important to notice that adult OMNI scale of perceived exertion has been validated also for elderly [5].
- In 2.3. Maximal Effort and Individual Ventilatory Threshold. Manufacturer's manual describes the calibration procedure of the flow meter (see page 109 of the Fitmate User manual, XIII Edition 05/2012) [6] and a specific sentence affirms “Regular calibration is necessary to ensure your system is acquiring reliable data. Flow/volume calibrations must be performed with a 3-liter calibration syringe.” Whereas for general purposes, it is possible to make the calibration procedure monthly, and for research purposes, a more frequent calibration is absolutely needed.
- In 2.4. Statistical Analysis. *F* values were added in Table 1.

In summary, we think that the results of our manuscript are based on scientific methodological methods. We evidenced that different obese conditions (obesity, sarcopenic obesity, or T2DM obesity) may induce different responses to submaximal exercise; thus, in order to prescribe the useful exercise intensity, it is important to assess the exercise capacity at the individual ventilatory threshold.

## Figures and Tables

**Table 1:** ANOVA results among obese groups (OB, OS, and T2DM).

	Num DF	Den DF	*F*	*P*
Variables at maximal effort				
Ventilation (L min^−1^)	2	45	1.54	0.23
$\dot{V}$O_2peak_ (mL kg^−1^ min^−1^)	2	45	0.66	0.52
$\dot{V}$O_2peak_ (mL min^−1^)	2	45	0.85	0.43
MET_peak_	2	45	0.65	0.53
%HR_max_	2	45	1.42	0.25
Variables at IVT				
$\dot{V}$O_2IVT_ (mL kg^−1^ min^−1^)	2	45	6.79	0.003
%VO_2peak_ (%)	2	45	9.34	<0.001
MET_IVT_	2	45	6.81	0.003
%HR_max_ (%)	2	45	6.41	0.004
%HRR_IVT_ (%)	2	45	6.86	0.003
ΔHR (bpm)	2	45	7.48	0.002

Note: OB: obese subjects; OS: sarcopenic obese subjects; T2DM: type 2 diabetes mellitus; $\dot{V}$O_2_: oxygen uptake; MET: metabolic equivalent; %HR_max_: percent of theoretical maximal heart rate; %HRR: percent of heart rate reserve.
